# Langerhans cell histiocytosis involving the liver of a male smoker: a case report

**DOI:** 10.1186/1752-1947-2-376

**Published:** 2008-12-08

**Authors:** Joana Savva-Bordalo, Margarida Freitas-Silva

**Affiliations:** 1 Department of Oncology, Portuguese Institute of Oncology Francisco Gentil, EPE, Porto, Portugal; 2 Department of Internal Medicine, Hospital de São João, EPE, Porto, Portugal; 3 Instituto Português de Oncologia Francisco Gentil; Serviço de Oncologia; Rua Dr António Bernardino de Almeida, 4200-072 Porto, Portugal

## Abstract

**Introduction:**

Langerhans' cell histiocytosis is a proliferative histiocytic disorder of unknown cause originating from dendritic cells.

**Case presentation:**

The authors report a case of Langerhans' cell histiocytosis in a 48-year-old man with multisystemic disease presentation, including liver involvement.

**Conclusion:**

Hepatic involvement is an uncommon feature in this rare disease and there is no consensus on the most effective therapeutic approach.

## Introduction

Langerhans' cell histiocytosis (LCH) belongs to a group of disorders where the common primary event is the accumulation and infiltration of monocytes, macrophages, and dendritic cells into the affected tissues. Its clinical presentation varies greatly, with symptoms ranging from mild to severe. The pathophysiology of LCH is not well understood and an optimal therapeutic strategy has yet to be established.

## Case presentation

A 48-year-old man was referred to our emergency room with a 10-day history of progressive dyspnea, non-productive cough and fever. He had previously visited his primary physician, who prescribed a 7-day treatment with an antibiotic. The patient had a 34-pack-year history of cigarette smoking, did not take any regular medications and had not recently visited any tropical country. Physical examination revealed hyperthermia (38.0°C), with blood pressure and pulse within normal ranges. He was slightly polypneic (28 cycles per minute) but had no other sign of respiratory difficulty. Respiratory sounds were diminished on both pulmonary bases and no adventitial sounds were heard. No rash or lymphadenopathy was noted and the remainder of his physical examination was normal. Room air arterial blood gas (ABG) was unremarkable and laboratory findings showed slight normocytic normochromic anemia (hemoglobin (Hb) 12.3 g/dL, hematocrit (Hct) 36.1%), leukocytosis (20,920/mm^3^), relative neutrophilia (75.5%), thrombocytosis (499,000/mm^3^) and elevated C-reactive protein (155.9 mg/L). Ionogram and renal function were normal. Chest X-ray revealed a mild reduction in lung volume and a mild and diffuse coarse reticular pattern on both lungs. The patient was diagnosed with community-acquired pneumonia, and, following admission to the Medicine ward, was started on empiric antibiotic therapy with levofloxacin.

**Figure 1:**
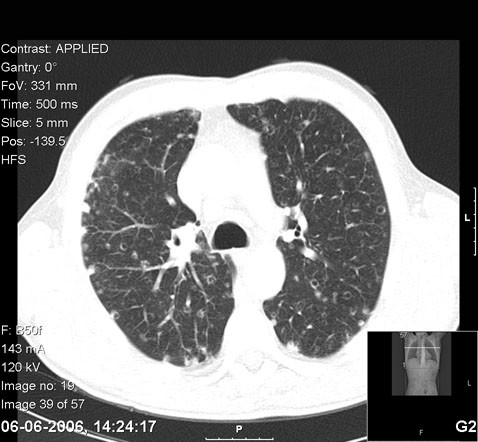
Thoracoabdominal-pelvic computed tomography scan of the patient, showing multiple cysts and nodules and interstitial thickening in the lungs.

**Figure 2:**
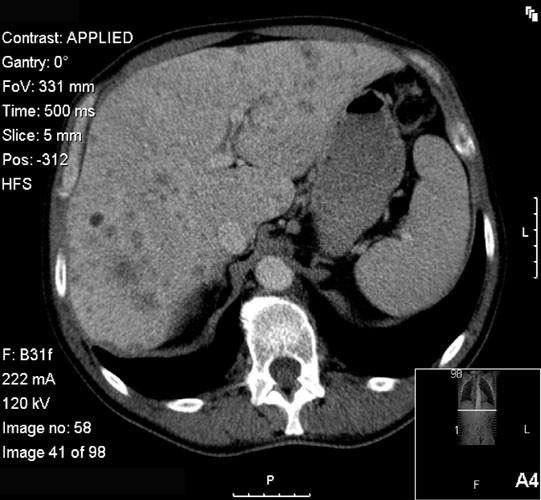
Thoracoabdominal-pelvic computed tomography scan showing several infracentimetric lymph nodes in the hepatic hilum.

**Figure 3:**
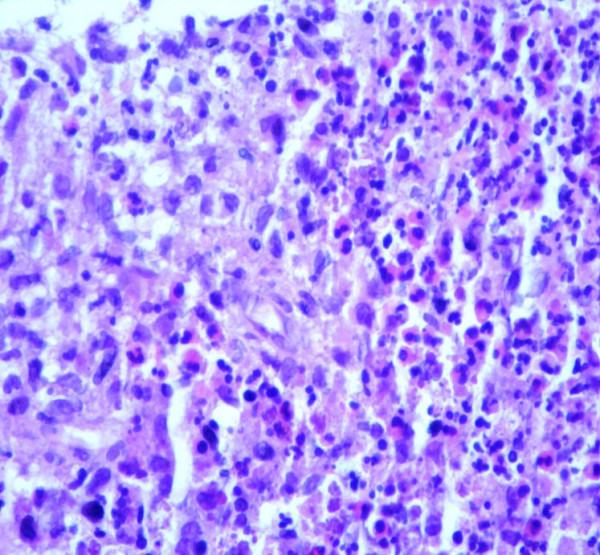
Histologic examination of a liver biopsy specimen.

During the first days after admission, persistent fever and high levels of inflammatory markers were noted. Given the patient's condition, an investigative diagnostic procedure was initiated. Blood cultures, HIV and hepatitis testing were negative. Coagulation, hepatic function and urine sediment were unremarkable. Bronchofibroscopy and bronchoalveolar lavage were negative for malignant cells, and virologic, bacterial, and mycological examinations and polymerase chain reaction were negative for mycobacterial DNA. Transthoracic echocardiography showed no evidence of any valvular vegetation, and a blood smear was not compatible with any myelodysplastic syndrome. Thoracoabdominal-pelvic computed tomography (CT) scan revealed several lymph nodes in all mediastinal compartments but no hilar adenomegalies. Multiple cysts and nodules, with mid to upper zone predominance, and interstitial thickening were observed in the lungs (Figure 1). The dimensions of the liver were enlarged, with several irregular hypoattenuating lesions and infracentimetric lymph nodes in the hepatic hilum (Figure 2). As a result, an ultrasound-guided liver biopsy was performed. Histologic (Figure 3) and immunohistochemical examination (i.e. positivity for S-100 protein and CD1a antigens) established a diagnosis of LCH.

A course of steroids (prednisolone, 1.0 mg/kg/day) was initiated, and the patient was encouraged to discontinue smoking immediately, which clearly improved the clinical course of the disease. Six months later, he remains asymptomatic, with low levels of inflammatory markers, although his lung and liver radiological patterns remain unchanged.

## Discussion

LCH is a proliferative histiocytic disorder of unknown cause originating from dendritic cells [1], with an estimated incidence of one to two cases per million population [2]. The disease most commonly occurs in individuals aged 21 to 69 years, with a mean age of 32 years [3]. From the number of involved organs, patients can be divided into two categories: those with isolated skin, lymph node, or bone lesions, and those with a disseminated form of LCH involving two or more organ systems, such as the lungs, liver and spleen [4]. Treatment of patients with LCH depends on the extent of the disease. Steroids may help to slow or even stop the progression of lung LCH and cessation of smoking is essential to prevent disease recurrence or progression [5]. Chemotherapeutic agents, such as vinblastine, methotrexate, cyclophosphamide, etoposide, and cladribine have been successful in patients with progressive disease unresponsive to corticosteroids and in those with multiorgan involvement [6]. Nevertheless, no systematic series of treatments for adults have been published and the optimal strategy has yet to be defined. In the case described here, however, steroid therapy plus the cessation of smoking improved the general condition of the patient.

## Conclusion

LCH is a rare disease with multiple clinical features, such that only histologic examination and immunohistochemical assays can lead to a final diagnosis. Smoking cessation and steroid therapy can improve the clinical course in patients with LCH and multisystem involvement. There is as yet no consensus on the ideal therapeutic regimen and management approach.

## Abbreviations

ABG: arterial blood gas; CT: computed tomography; Hb: hemoglobin; Hct: hematocrit; LCH: Langerhans' cell histiocytosis.

## Consent

Written informed consent was obtained from the patient for publication of this case report and any accompanying images. A copy of the written consent is available for review by the Editor-in-Chief of this journal.

## Competing interests

The authors declare that they have no competing interests.

## Authors' contributions

JSB and MFS dealt directly with the patient, contributed to the writing and editing of the manuscript. Both authors read and approved the final manuscript.
